# Novel compound heterozygous *CCDC40* mutations in a familial case of primary ciliary dyskinesia

**DOI:** 10.3389/fped.2022.996332

**Published:** 2022-09-29

**Authors:** Liqing Zhao, Suqiu Huang, Wei Wei, Bingyao Zhang, Wenxiang Shi, Yongzhou Liang, Rang Xu, Yurong Wu

**Affiliations:** ^1^Department of Pediatric Cardiology, Xinhua Hospital Affiliated to Shanghai Jiao Tong University School of Medicine, Shanghai, China; ^2^Department of Pediatrics, Chengdu Second People’s Hospital, Chengdu, China; ^3^Scientific Research Center, Xinhua Hospital Affiliated to Shanghai Jiao Tong University School of Medicine, Shanghai, China; ^4^Department of Cardiology, Shanghai Children’s Hospital, Shanghai Jiao Tong University School of Medicine, Shanghai, China

**Keywords:** primary ciliary dyskinesia, *CCDC40*, situs inversus, annular pancreas, pathogenic mutation

## Abstract

Primary ciliary dyskinesia (PCD) is a rare genetic disorder characterized by motile ciliary dysfunction and impaired ultrastructure. Despite numerous studies, the genetic basis for about 30% of PCD cases remains to be elucidated. Here, we present the identification and functional analysis of two novel mutations in the gene encoding coiled-coil domain-containing protein 40 (CCDC40), which are found in a familial case of PCD. These novel *CCDC40* mutations, NM_017950.4: c.2236-2delA and c.2042_2046delTCACA, NP_060420.2: p.(Ile681fs), were identified by whole-exome sequencing (WES). Sanger sequencing was then performed to confirm the WES results and determine the *CCDC40* gene sequences of the proband’s parents. The c.2042_2046delTCACA mutation disrupts the reading frame of the protein and is therefore predicted to produce a non-functional protein. Using a minigene assay with the pcDNA3.1(+) plasmid, we further investigated the potential pathogenic effects of the c.2236-2delA mutation and found that this mutation leads to formation of a truncated protein *via* splicing disruption. Thus, in summary, we identified two mutations of the *CCDC40* gene that can be considered pathogenic compound heterozygous mutations in a case of familial PCD, thereby expanding the known mutational spectrum of the *CCDC40* gene in this disease.

## Introduction

Primary ciliary dyskinesia (PCD) (OMIM#244400) is a rare genetic disorder affecting between 1:15,000 and 1:30,000 individuals worldwide (1, 2). Most PCD cases are inherited in an autosomal recessive manner, although rare cases have been reported to show X-linked inheritance (2, 3). The disease is characterized by impaired function of motile cilia (4), and clinical manifestations include chronic or recurrent respiratory tract infections, situs inversus (SI), conductive hearing impairment, and infertility (5). In addition, dextrocardia with SI, involving a total mirror-image arrangement of all thoracic and abdominal viscera, occurs in approximately 50% of PCD cases, and these were shown to be diagnosed earlier than cases with normal organ position (6).

To date, a series of monogenic mutations in over 40 causative genes that contribute to the abnormal structure and function of motile cilia in PCD has been reported (7). The guidelines agree that either a biallelic pathogenic mutation or hemizygous X-linked mutation in a known PCD gene is sufficient to confirm a diagnosis (8, 9). PCD causative genes have been shown to affect ciliary protein function and transport, as well as docking of ciliary structures. In particular, most affected individuals exhibit mutations in genes that encode dynein arm components, such as dynein axonemal heavy chain (DNAH)5, DNAH11, dynein axonemal intermediate chain (DNAI)1, DNAI2, and thioredoxin domain-containing (TXNDC)3 (7). Coiled-coil domain-containing protein (CCDC) 39 and *CCDC40*, the 96-nm ruler proteins, are responsible for the correct establishment of 96-nm repeats along the ciliary axoneme (7). *CCDC40* is also thought to be essential for the docking of inner dynein arms (IDAs) and plays a vital role in left–right axis specification (10). However, despite numerous genetic findings, the genetic basis for about 30% of PCD cases remains unknown, suggesting the existence of other causative genes and mutations.

In this study, we report the genetic analysis of a newborn who was prenatally diagnosed with dextrocardia, total SI, and duodenal obstruction. The proband also exhibited neonatal respiratory distress at term birth. Diagnosis of PCD was confirmed according to guidelines of the American Thoracic Society (9), and exome analysis of the proband identified novel heterozygous mutations, NM_017950.4: c.2236-2delA and c.2042_2046delTCACA, NP_060420.2: p.(Ile681fs), in the *CCDC40* gene. These two novel *CCDC40* mutations were inherited from the proband’s mother and father, respectively, as confirmed by Sanger sequencing. Using a series of bioinformatic analyses and the minigene assay, we further verified the pathogenicity of the mutated sites. Thus, to the best of our knowledge, our study is the first to report a pathogenic role for these heterozygous *CCDC40* gene mutations in PCD.

## Materials and methods

### Subjects and clinical examinations

**FIGURE 1:**
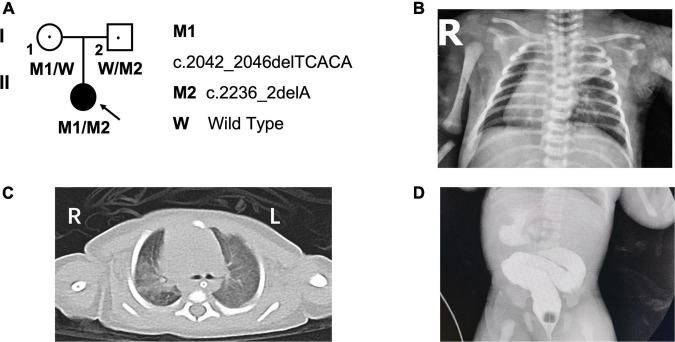
Pedigree of the family and the clinical photographs of the proband. **(A)** The pedigree of the patient’s family. **(B)** The chest radiography and **(C)** computed tomography scan indicated situs inversus and bronchopneumonia. **(D)** The gastrointestinal contrast indicated duodenal obstruction. L, left; R, right; “blue arrow” showing bronchopneumonia; “red arrow” showing the location of duodenal obstruction.

A Chinese family, including the proband, was analyzed in this study (Figure 1A). The proband was transferred to the neonatal intensive care unit (NICU) immediately after birth due to prenatal suspected gastrointestinal tract anomalies and SI in the third trimester. A series of examinations were performed after birth, including chest radiography, echocardiography, chest computed tomography scan, and gastrointestinal contrast (Figure 1). This study was conducted in compliance with the Declaration of Helsinki and was approved by the Ethics Committee of Xinhua Hospital (Approval no. XHEC-QT-2021-042); written informed consent was obtained from both parents.

### Whole-exome sequencing

Genomic DNA (gDNA) was isolated from 2-ml peripheral blood samples obtained from each family member using the QIAamp DNA Mini Kit, according to the manufacturer’s instructions (QIAGEN, Hilden, Germany). Purified gDNA from the proband was analyzed by whole-exome sequencing (WES), using a commercial sequencing services company (Shanghai Biotechnology Co., Ltd., Shanghai, China). Exome capture was performed using the SureSelect Human All Exon V6 system (Agilent Technologies, Santa Clara, CA, USA), according to the manufacturer’s instructions. Exome sequencing libraries were then subjected to quality control testing and analyzed by 2 × 150 bp paired-end sequencing on the HiSeq X Ten platform (Illumina, San Diego, CA, USA). FASTQ files were aligned to the human reference genome (hg19/GRCh37). Based on the quality of the reads, the fragments were analyzed with different tools (11), and the detected variants were exported in a Microsoft Excel file.

### Mutational confirmation with Sanger sequencing

To confirm WES results in the proband and determine the *CCDC40* gene sequences of the proband’s parents, we performed bidirectional Sanger sequencing, using an ABI 3730 capillary sequencing instrument (Applied Biosystems, Foster City, CA, USA). Primer sequences are listed in Table 1.

**TABLE 1:** Primer sequences for Sanger sequencing and minigene assays.

Primer name	Primer-F (5′–3′)	Primer-R (5′–3′)
CCDC40-Mother	ACAAGTAGGT CCCTTGCTGG	TGACTTAGAG CGCTTGGCAC
CCDC40-Father	TCCAGAGCCC CTTAGCCATA	GCTTGGAGCC ATTTCGTGAC
CCDC40-131415-cDNA	GACCACCCTG GACATCACAC	GCGCACGAAC TCATTCTCTG

### Bioinformatics analysis

We used multiple bioinformatics programs, including the Human Splicing Finder (HSF) tool and MutationTaster, to predict possible impacts of the detected *CCDC40* variants.

### Minigene assays

We used the minigene assay to assess the pathogenicity of the c.2236-2delA mutation and determine the splicing capacity of the wild-type (WT) and mutant sequence (12). Because the c.2236-2delA mutation is in intron13, we amplified 2,962-bp gDNA fragments that include exon13, intron13, exon14, intron14, and exon15 from the PCD patient and a control patient (primer sequences are listed in Table 1). To construct *CCDC40* expression plasmids, the WT and mutant PCR products were inserted into the pcDNA3.1(+) vector (Invitrogen, Thermo Fisher Scientific, Waltham, MA, USA) by the GENEWIZ company, using the Seamless Cloning Kit (Hanbio, China). The WT construct was named WT-E131415-pcDNA3.1, and the mutant construct was named MUT-E131415-pcDNA3.1. After plasmid amplification in DH5α *Escherichia coli* and plasmid purification (Omega Bio-Tek, Norcross, GA, USA), the sequences and correct orientations of all plasmid constructs were validated by Sanger sequencing, as described above.

### Cell culture and transfection

Human embryonic kidney 293T cells were maintained in Dulbecco’s Modified Eagle Medium (DMEM) with high glucose (HyClone, Logan, UT, USA) and 10% fetal bovine serum (FBS; Gibco, Thermo Fisher Scientific). Cells were incubated at 37°C in a humidified atmosphere with 5% CO_2_ and plated onto 6-well plates for 18 h before transfection at a confluence of 60–70%. WT and mutant *CCDC40* plasmids were transfected into 293T cells using FuGene HD Transfection Reagent (Promega, Madison, WI, USA), according to the manufacturer’s protocol.

### RNA extraction and reverse transcription-PCR

Total RNA was extracted from transfected 293T cells using TRIzol Reagent (Invitrogen, Thermo Fisher Scientific), and RT-PCR was performed with the PrimeScript RT Reagent Kit (TaKaRa Bio, Kusatsu, Shiga Prefecture, Japan). Primers for PCR amplification are listed in Table 1.

## Results

### Clinical findings

The proband (G1P1), a female infant, is the first child of non-consanguineous and healthy parents. She was born at 39-weeks’ gestation to a 28-year-old mother and a 27-year-old father, and her family history is unremarkable. Postnatal transthoracic echocardiography revealed a diagnosis of mirror-image dextrocardia, interrupted inferior vena cava (IVC), secondary atrial septal defect (ASD), and a patent foramen ovale (PFO). Chest radiograph (Figure 1B) and computed tomography scan further indicated bronchopneumonia and total SI (Figure 1C), and gastrointestinal contrast confirmed a diagnosis of duodenal obstruction (Figure 1D). Lastly, the patient received a diagnosis of annular pancreas based on results of surgery performed on the second day after birth.

### Mutation identification by exome analysis and Sanger sequencing

**FIGURE 2:**
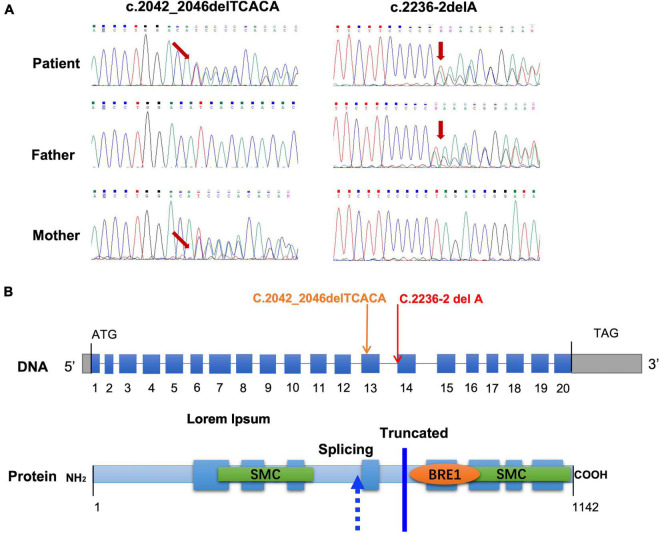
Identification and analysis of mutations in the CCDC40 gene. **(A)** Sanger sequencing chromatogram of the mutations in CCDC40 from the patient and the parents of the patient. **(B)** Location of the mutations at the nucleotide and protein level. The blue boxes represent the 20 coding exons and the gray boxes represent 5′ and 3′ untranslated regions.

To identify possible pathogenic mutations, we performed WES analysis of a gDNA sample from the proband, yielding approximately 6 Gb data and producing 205,579,454 total reads. From this analysis, we obtained a coverage of 1x, 20x, and 50x across 99.38, 93.93, and 85.23%, respectively, of the captured target area. We first filtered the WES data by (1) including only variants located in exonic or splicing regions, (2) excluding synonymous variants, and (3) excluding variants with a minor allele frequency (MAF) > 0.001. Next, based on the observed clinical phenotypes and autosomal recessive inheritance patterns, we identified two mutations of the *CCDC40* gene, c.2236-2delA and c.2042_2046delTCACA p.(Ile681fs), that may function as pathogenic compound heterozygous mutations in PCD. We then performed Sanger sequencing, which confirmed the presence of these variants in the proband and revealed that the frameshift mutation c.2042_2046delTCACA was inherited from the mother, whereas the c.2236-2delA mutation was inherited from the father (Figure 2A). Copy number variation (CNV) analysis was also performed on WES data, and no abnormal CNV was detected.

### Prediction of mutation pathogenicity

We next predicted the pathogenicity of the frameshift mutation c.2042_2046delTCACA (p.Ile681fs) and the splicing mutation c.2236-2delA in *CCDC40* (Figure 2B) using two online analysis tools: MutationTaster^1^ and HSF.^2^ MutationTaster classified the c.2042_2046delTCACA mutation as “Disease-causing,” based on the prediction that it causes a frameshift and creates a premature stop codon p.(Ile681fs), leading to translation of a truncated protein without the N-terminal 390 amino acids. Results of HSF analysis further showed that the c.2236-2delA mutation likely affects splicing by altering the WT acceptor splice site, with the variation (%) of Position Weight Matrices relative to the WT site (–30.27) indicating that this site is dysfunctional in mutant mRNA. Thus, our results strongly suggest that both mutations in the *CCDC40* gene are pathogenic. Furthermore, these variants are novel, as neither has been reported in the 1,000 Genomes Project database, the Exome Aggregation Consortium (ExAC) database, or the Human Gene Mutation Database (HGMD).

### Minigene assay

**FIGURE 3:**
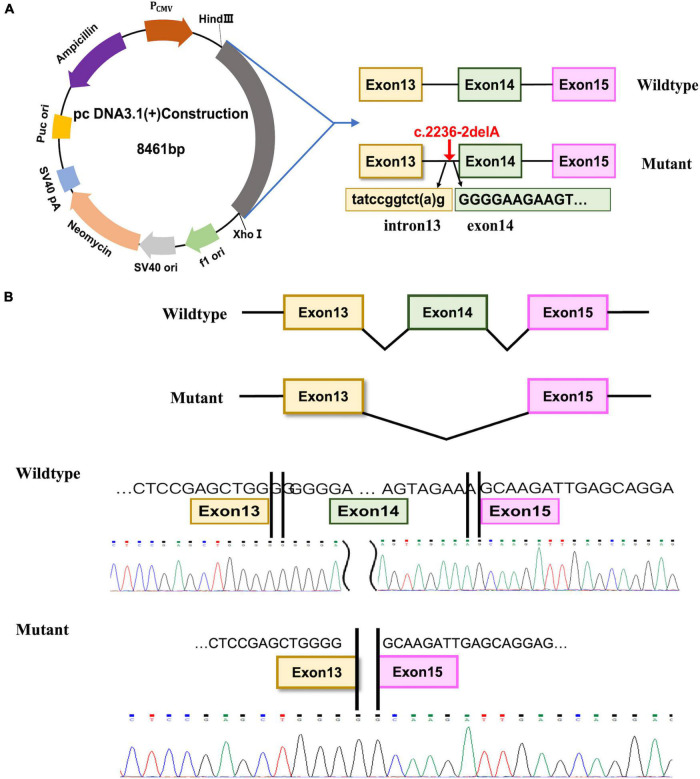
Minigene assay and Sanger sequencing of spliced transcripts. **(A)** Diagram of the DNA, the location of c.2236-2delA mutation and the fragment inserted into the pcDNA3.1(+) plasmid. **(B)** The spliced sequences and Sanger sequencing chromatogram of the transcripts from the wide-type and the mutated construct.

Lastly, we performed a minigene assay with the pcDNA3.1(+) plasmid to investigate the potential pathogenic effect of the c.2236-2delA mutation. To this end, RNA was extracted from 293T cells transfected with WT-E131415-pcDNA3.1 or MUT-E131415-pcDNA3.1 (Figure 3A), and after reverse transcription (RT) and amplification, the spliced sequences were examined using Sanger sequencing. Our results revealed that the WT construct produces a whole 630-bp band, which results from normal splicing between exon13 and exon15 (Figure 3B). However, the mutant produces a band of 416 bp, resulting from exon14 skipping between exon13 and exon15 (Figure 3B) and leading to a frameshift in the transcript. These results therefore indicate that the c.2236-2delA mutation results in production a truncated protein by disrupting splicing.

## Discussion

In the present study, we performed genetic analysis of a newborn PCD patient presenting with neonatal respiratory distress, secondary ASD, SI with dextrocardia, and annular pancreas. WES revealed two novel mutations, c.2236-2delA and c.2042_2046del TCACA, p.(Ile681fs), in the *CCDC40* gene. These two mutations carried by the proband were inherited from the father and mother, respectively, which generated compound heterozygosity in the patient.

Diagnosis of PCD is often delayed due to the non-specific symptoms and lack of early diagnostic methods. Studies have shown that different ciliary phenotypes in PCD patients result from a variety of mutations in cilia-related genes, and genotype–phenotype relationships underlying this disease are becoming more apparent (13). Genetic testing is therefore useful to confirm the genotype of probable PCD cases and is an important component of PCD diagnosis based on to current guidelines (8, 9), with a PCD genetic testing panel currently in development. In particular, genetic analysis is critical for early diagnosis, determination of proper disease-management strategies, and family genetic counseling for PCD cases (7, 8).

*CCDC40* belongs to a group of evolutionarily conserved coiled-coil domain-containing proteins that, together with *CCDC39*, is known as a 96-nm ruler protein, due its essential role in ensuring the correct establishment of 96-nm repeats along the ciliary axoneme (7). *CCDC40* also appears to be required for axonemal recruitment of *CCDC39* (10). The *CCDC40* protein, which contains 1,142 residues and eight predicted coiled coils, is specifically expressed in the embryonic node and is essential for the formation and maintenance of cilia (14) (Figure 2B). The coiled-coil motif is a common structural domain in eukaryotic and prokaryotic proteins and can act as an adapter between molecules (14). *CCDC40* also contains a conserved BRE1 domain of unknown function, as well as two large Structural Maintenance of Chromosomes (SMC) conserved domains, which are found in several ciliary proteins and likely play a role in microtubule-based ciliary transport processes (15) (Figure 2B).

A number of mutations in *CCDC40* have been identified as novel candidates for gene testing in PCD patients, with these mutations estimated to cause approximately 4–8% of PCD cases (16). Previous studies in animal models have suggested that mutations in *CCDC40* disrupt cilia function *via* the loss of IDAs, which is accompanied by variably expressed disorganization of the 9 + 2 microtubule arrangement (15). These CCDC40-affected cilia often show loss of waveform motion and become completely immotile (15). Here, we found that the c.2236-2delA *CCDC40* gene variant is predicted to affect the acceptor splice site, and results of our minigene assay confirm this mutation causes exon skipping, which results in production of abnormal *CCDC40* mRNA with an altered reading frame. The other variant, c.2042_2046delTCACA, directly shifts the reading frame and introduces a stop codon, thereby putatively leading to production of a truncated, non-functional protein. Notably, both of these variants result in complete loss of the second SMC domain in the *CCDC40* protein (Figure 2B), which may explain the observed PCD phenotype in the proband.

PCD patients with *CCDC40* mutations or those displaying associated ultrastructural defects (inner dynein arm/central apparatus/microtubular defects) have been reported to show worse lung disease and poorer growth compared to those with outer dynein arm defects (defined by ultrastructure and mutations in associated genes) (4). In addition, the long-term prognosis of *CCDC39*- or *CCDC40*-affected cases is worse than for other PCD patients, with similar findings reported for individuals with cystic fibrosis, as well (4). In both instances, recurrent respiratory tract infections in patients with dysfunctional cilia may lead to irreversible lung damage. Early diagnosis is therefore important to select an appropriate disease-management strategy for preventing the onset of this damage.

Approximately half of all PCD patients exhibit SI, also referred to as Kartagener syndrome (KTS) (17), and this trait appears to result, in part, from random determination. However, one previous study found that PCD patients with SI show more complex genetic heterogeneity than unaffected individuals (18), and the disease mechanisms affecting nodal cilia function are also important for determination of left–right asymmetry during embryogenesis. Of note, *CCDC40* in particular, was shown to be expressed in the embryonic node and midline tissues in a mouse model of PCD (10). Thus, the observed phenotype of total SI with mirror-image dextrocardia in the proband is consistent with these genetic findings.

Annular pancreas, another phenotype present in the proband for our study, is a rare congenital gastrointestinal anomaly characterized by a ring of pancreatic tissue surrounding the descending portion of the duodenum. It is thought to originate from incomplete rotation of the ventral pancreatic bud, and many reports have indicated a genetic basis for this anomaly (19). However, to the best of our knowledge, our study is the first showing annular pancreas associated with PCD, and thus, the genetic connection underlying this feature requires further investigation.

## Conclusion

In conclusion, we identified two compound heterozygous mutations in the *CCDC40* gene of a newborn baby with PCD, one affecting the acceptor splice site and one causing a direct frameshift, both of which are reported for the first time in this study. These results provide new insight into the genetic basis of PCD and will be critical for obtaining a definitive diagnosis, determining a follow-up disease-management strategy, and providing comprehensive genetic counseling for this family.

## Data availability statement

The datasets presented in this study can be found in online repositories. The names of the repository/repositories and accession number(s) can be found in the article/Supplementary material.

## Ethics statement

The studies involving human participants were reviewed and approved by the Ethics Committee of Xinhua Hospital. Written informed consent to participate in this study was provided by the participants’ legal guardian/next of kin.

## Author contributions

YW, RX, and LZ: study concept and research design. LZ, BZ, and WS: patient’s clinical data. SH, WW, and YL: sequencing and mutation validation. LZ, SH, and YW: writing of the manuscript. All authors have read and agreed to the published version of the manuscript.

## Footnotes

1
http://www.mutationtaster.org/

2
http://www.umd.be/HSF3/HSF.shtml/

## Abbreviations

PCD: Primary ciliary dyskinesia
SI: situs inversus
*CCDC40*: coiled-coil domain-containing protein 40
DNAH: dynein axonemal heavy chain
DNAI: dynein axonemal intermediate chain
TXNDC: thioredoxin domain-containing
IDAs: inner dynein arms
NICU: neonatal intensive care unit
WES: whole-exome sequencing
gDNA: Genomic DNA
HSF: Human Splicing Finder
WT: Wide-type
IVC: inferior vena cava
ASD: atrial septal defect
PFO: patent foramen ovale
CNV: Copy number variation
SMC: Structural Maintenance of Chromosomes
KTS: Kartagener syndrome.
